# Overexpression of exogenous *biuret hydrolase* in rice plants confers tolerance to biuret toxicity

**DOI:** 10.1002/pld3.290

**Published:** 2020-11-29

**Authors:** Kumiko Ochiai, Asuka Uesugi, Yuki Masuda, Megumi Nishii, Toru Matoh

**Affiliations:** ^1^ Graduate School of Agriculture Kyoto University Kyoto Japan; ^2^ Kyoto Agriculture Research Institute (Kyoto Nogyo no Kenkyusho) Kyoto Japan

**Keywords:** biuret toxicity, fertilizer, nitrogen, rice, urea

## Abstract

Biuret, a common impurity in urea fertilizers, is toxic to plants, but little is known about the physiological mechanisms underlying its toxicity. Here, we analyzed biuret toxicity in rice (*Oryza sativa*) plants. We carried out uptake experiments using ^15^N‐labelled biuret and demonstrated that biuret could reach sub millimolar concentrations in rice plants. We also demonstrated that the hydrolysis of biuret in plant cells could confer biuret tolerance to rice plants. This occurred because transgenic rice plants that overexpressed an exogenous *biuret hydrolase* cloned from a soil bacterium gained improved tolerance to biuret toxicity. Our results indicate that biuret toxicity is not an indirect toxicity caused by the presence of biuret outside the roots, and that biuret is not quickly metabolized in wild‐type rice plants. Additionally, it was suggested that biuret was used as an additional nitrogen source in transgenic rice plants, because *biuret hydrolase*‐overexpressing rice plants accumulated more biuret‐derived N, as compared to wild‐type rice.

## 1 INTRODUCTION

Urea, currently the most widely used nitrogen (N) fertilizer worldwide (IFASTAT, https://www.ifastat.org/databases), might contain biuret [(CONH_2_)_2_NH], as a common impurity. Biuret is formed by the thermal condensation of urea. It has been known since the 1950s that excessive amounts of biuret in urea fertilizers cause injury in crops (Jones, 1954; Sanford et al., 1954). A wide range of crops can be potentially affected by biuret toxicity, which often manifests as leaf chlorosis and stunted growth, especially in the young seedling stage (Mikkelsen, 1990). Earlier studies indicated that biuret inhibited protein synthesis in *Xanthium pensylvanicum* leaves (Webster et al., 1957) and wheat (*Triticum aestivum*) germplasms (Ogata & Yamamoto, 1959). The protein content, however, did not so much decrease in biuret‐injured orange (*Citrus sinensis*) leaves (Impey & Jones, 1960). It remains uncertain whether biuret has a direct effect on the protein synthetic machinery. Additionally, ultrastructural analyses showed that changes in chloroplast structure in biuret‐injured leaves were similar to those in senescent leaves in grapefruit (*Citrus paradise*) and orange plants (Achor & Albrigo, 2005). Moreover biuret seems to remain unmetabolized in plants, because it was still detected in orange leaves, eight months after foliar spraying was performed (Impey & Jones, 1960). The exact mechanism underlying biuret toxicity in plants, however, is still far from being understood.

To avoid this hazard, the biuret content in fertilizers is regulated; for example, the upper limit of biuret‐N in urea fertilizer is set at 2% of the total N content in Japanese law. Currently, biuret injury has become less frequent in farmers’ fields, owing to advances in the technology used for manufacturing urea fertilizers. One method for the fertilization of rice (*Oryza sativa*) crops involves a single basal application of polymer‐coated urea into seedling trays, which improves N use efficiency and labor efficiency. The extremely high density of coated urea fertilizer adjacent to the roots resulted in biuret toxicity, even though fertilizers that met the official standards were used (Tanahashi et al., 2003). This illustrates that the risk of biuret toxicity in crops remains latent.

Certain soil bacteria decompose biuret (Aukema et al., 2020; Cameron et al., 2011; Esquirol et al., 2018, 2020; Jensen & Schrøder, 1965; Martinez et al., 2001; Robinson et al., 2018). Biuret in soil occurs as an impurity of urea fertilizer. It is also produced by the degradation of cyanuric acid, an intermediate metabolite of *s*‐triazine compounds such as melamine and atrazine (Aukema et al., 2020). The evolution of biuret catabolizing enzymes in bacteria is considered to be associated with the use of urea fertilizer and *s*‐triazine herbicide (Esquirol et al., 2020; Robinson et al., 2018). One molecule of biuret is converted into three ammonium and two bicarbonate ions via the biodegradation pathway. The first step of biuret degradation is the hydrolysis of biuret into ammonium and allophanate, which is catalyzed by biuret hydrolase (Cameron et al., 2011). Then, allophanate could either undergo spontaneous decarboxylation to form urea under neutral and acidic conditions, or be hydrolyzed further by allophanate hydrolase into ammonium and bicarbonate (Cheng et al., 2005).

Homologs of *biuret hydrolase* were detected in a broad range of microorganisms but remained undetected in animals and land plants (Robinson et al., 2018). Although it is not known yet how much biuret plants would take up and accumulate, introduction of *biuret hydrolase* from soil bacteria might contribute to biuret detoxification in plant cells. Furthermore, biuret could act as a slow‐release N fertilizer as researchers have suggested (Esquirol et al., 2020; Sahrawat, 1981) and as a weed controller (Figure S1) if we could confer the biuret‐detoxifying ability to crop plants.

Here, we investigated biuret injury in rice plants. We first evaluated biuret uptake in rice plants quantitatively using ^15^N‐labelled biuret. Furthermore, we generated transgenic rice plants that overexpressed bacterial *biuret hydrolase* and examined their biuret tolerance. Additionally, we performed a microarray‐based transcriptome analysis using suspension rice cells to investigate the general effects of biuret on rice cells.

## 2 MATERIALS AND METHODS

### 2.1 Plant materials and growth conditions

Seeds of Nipponbare, a *japonica* rice (*Oryza sativa*) cultivar, were purchased from Nouken Inc. (Kyoto, Japan). Transgenic rice lines overexpressing bacterial *biuret hydrolase,* which were generated from Nipponbare in this study, were self‐pollinated to obtain T_1_ progenies. T_1_ and T_2_ seeds were used for experiments.

Rice plants were grown under hydroponic conditions in a growth chamber (NS‐280 FHW; Takayama Seisakusyo, Kyoto, Japan) under a temperature, photo period, and light intensity of 30ºC, 12 hr, and 350 mmol/m^2^ s^−1^, respectively. Rice seeds were soaked in distilled water supplemented with fungicide (Trifumin; Nippon Soda Co., Ltd., Tokyo, Japan) for three days at 30ºC. Ten or twelve of the imbibed seeds were sown on a nylon‐mesh (18 mesh, 24 × 36 mm) supported by a plastic frame floating on the culture solution. The culture solution contained 1 mmol/L (NH_4_)_2_SO_4_, 0.25 mmol/L KH_2_PO_4_, 0.5 mmol/L KCl, 0.5 mmol/L CaCl_2_, 0.5 mmol/L MgCl_2_, 0.09 mmol/L ethylenediamine‐N,N,N',N'‐tetra acetic acid, iron(III), sodium salt, trihydrate (FeNa‐EDTA) and Arnon's micronutrient (cited by Hewitt, 1966). The culture solutions were not aerated, and the solutions were renewed once a week. Seeds that did not germinate were removed from the mesh at an appropriate time. Biuret was included in the culture solution whenever necessary.

Rice Oc cell suspension culture line (Baba et al., 1986) was provided by the RIKEN BRC, participating in the National BioResource Project of the MEXT/AMED, Japan. The suspension cells were maintained shaking in the dark at 25ºC in 80 ml of Murashige and Skoog medium with 3% sucrose and 1 mg/L 2,4‐dichlorophenoxyacetic acid, pH 5.7 in a 300‐mL conical flask and subcultured weekly. When necessary, filter‐sterilized biuret solution was added to the autoclaved medium. Suspension cells were exposed to biuret toxicity by subculturing 2 ml of seven‐day‐old cell suspension into 80 ml of the medium supplemented with biuret at desired concentrations. At harvest, cells in a flask were collected by suction filtration.

### 2.2 Measurement of biuret in culture solutions

Aliquots (0.94 ml) of the culture solution were mixed with 10 µl of 0.5 mol/L potassium phosphate buffer (pH 6.8) and 50 µl of methanol, and filtered using the Cosmospin Filter G (0.2 µm; Nacalai tesque, Kyoto, Japan). A 20‐µl aliquot of the sample was injected into an HPLC system (LC‐10AS, Shimadzu, Kyoto, Japan) equipped with a COSMOSIL 5C18‐PAQ column (5 µm, 4.6 mm I.D. × 250 mm; Nacalai tesque, Kyoto, Japan). The eluent consisted of 5 mmol/L potassium phosphate buffer (pH 6.8) and 5% (v/v) methanol, and the flow rate was 0.2 ml/min. The absorbance of the eluate was monitored at 210 nm using a UV detector (SPD‐10A, Shimadzu, Kyoto, Japan).

### 2.3 Determination of biuret uptake in rice plants

^15^N‐labelled biuret (biuret‐^15^N_3_, ≥98 atom% ^15^N) was purchased from Sigma‐Aldrich Co (St. Louis, MO, USA). Rice seedlings were raised in the absence of biuret for 17 days. Then, seedlings with uniform sizes were picked from the mesh, and two plants were transferred to a 50‐mL glass vial with 40 ml of culture solution, one day before performing the uptake experiments, to eliminate the possible effects of damaged roots. When we used transgenic lines, the presence of the transgene was confirmed beforehand by PCR, using the DNA extracted from leaf blades of the second leaf. Uptake experiments were initiated by the addition of an aliquot of ^15^N‐labeled biuret solution into the vial, to achieve a solution with a final biuret concentration of 0.3 mmol/L. Plants were allowed to take up ^15^N‐labeled biuret for 48 hr. Control plants, to which biuret was not applied, were also grown similarly.

At harvest, roots were rinsed twice in 100 ml of distilled water (3 min each). Plants were separated into shoots and roots, dried in an oven at 70ºC for two days, and ground into a powder using a ball mill. The N concentration in plant samples was determined using a CN analyzer (SUMIGRAPH NC‐22F, Sumika Chemical Analysis Service, Osaka, Japan). The atom % of ^15^N was determined under contract (Shoko Science Co Ltd, Kanagawa, Japan). Biuret‐derived N content in plant parts were calculated from the ^15^N content in samples.

### 2.4 Generation of transgenic rice plants overexpressing *biuret hydrolase*

Transgenic Nipponbare plants that over‐expressed bacterial *biuret hydrolase* under the control of the cauliflower mosaic virus 35S promoter were generated. A biuret decomposing soil bacterium, *Rhizobium* sp. KaB01, which was isolated in this study, was used as a *biuret hydrolase* donor (see Supplemental methods). Insert DNA was amplified from the genomic DNA of KaB01 via PCR, using Prime Star polymerase (Takara Bio, Shiga, Japan) and primers 5′‐CACCATGAAGACACTTTCCAGCGC‐3′ and 5′‐TGGCAAATGCCTCTCAAGG‐3′. Subcloned PCR products in the vector pENTR/D‐TOPO (Life Technologies, Carlsbad, CA) were then transferred to a binary vector pGWB502omega (Nakagawa et al., 2007), via the LR reaction. The transformation of rice was mediated by an agrobacterium, as described by Toki et al. (2006), using the *Agrobacterium tumefaciens* strain EHA105. The presence of the transgene in regenerated T_0_ plants was confirmed by PCR, using the Blend Taq polymerase (Toyobo, Osaka, Japan) and primers 5′‐ATGAAGACACTTTCCAGCGC‐3′ and 5′‐TGGCAAATGCCTCTCAAGG‐3′.

### 2.5 Transgene expression analysis

Total RNA was extracted from the youngest leaf blade of regenerated T_0_ plants at their vegetative growth stage, using the Plant Total RNA Extraction Miniprep System (Viogene, Taipei, Taiwan). First‐strand cDNA was synthesized from total RNA using an oligo dT primer and ReverTra Ace polymerase (Toyobo, Osaka, Japan). Quantitative real‐time RT‐PCR was performed in duplicate with the TP850 thermal cycler dice real time system single (Takara Bio, Shiga, Japan), using the THUNDERBIRD^®^ SYBR qPCR Mix (Toyobo, Osaka, Japan) and primers 5′‐AGCCGATCAAAAAGGTGCTGTC‐3′ and 5′‐AATGATATCCCAGCCAGGTTCTCC‐3′. The relative expression level was calculated as a ratio to a geometric mean of the expression of *ubiquitin* and *actin*. The sequences of primers were 5′‐AGAAGGAGTCCACCCTCCACC‐3′ and 5′‐GCATCCAGCACAGTAAAACACG‐3′ for *ubiquitin* and 5′‐ATCCTTGTATGCTAGCGGTCGA‐3′ and 5′‐ATCCAACCGGAGGATAGCATG‐3′ for *actin*.

### 2.6 Assay for biuret hydrolase activity

Crude plant extracts were prepared as described below. Shoots of individual 19‐day‐old wild‐type Nipponbare and transgenic B3‐9‐1 plants and 16‐day‐old B2‐3‐3‐3 plants were weighed, and ground into powders under liquid nitrogen using a mortar and pestle. Then, the powdered tissue was homogenized in ten volumes of 25 mmol/L 3‐(*N*‐morpholino) propanesulfonic acid (MOPS) buffer (pH 8.0). After centrifugation, the supernatant was used for the enzyme assay. Extractions were performed in duplicate for transgenic lines, and in triplicate for wild‐type plants.

The biuret hydrolase assay was carried out as described by Martinez et al. (2001). The assay solution contained 50 mmol/L sodium phosphate buffer (pH 8), 3 mmol/L biuret, and crude cell extracts. The assay mix was incubated at 30ºC, and the reaction was stopped by the addition of 0.5 mol/L H_2_SO_4_. The amount of ammonia released from biuret was colorimetrically determined by indophenol blue method (Weatherburn, 1967). Protein concentrations were determined by the Bradford method, using Protein Assay CBB Solution (Nacalai tesque, Kyoto, Japan). Measurements were performed in duplicate.

### 2.7 Evaluation of biuret tolerance in transgenic rice plants over expressing *biuret hydrolase*

Seeds from each line were sown onto two nylon‐mesh floats on the culture solution in the absence of biuret and grown for two days, to achieve uniform germination. Then, one of the two floats were transferred into a new 2‐L container that did not contain biuret and the other was transferred into another 2‐L container containing 0.3 mmol/L biuret. Plants were harvested 7 days after the onset of the biuret treatment. After determining plant heights, leaf blades from the third leaf were used to determine the chlorophyll content, and leaf blades from the second leaf were used for DNA extraction. Chlorophyll was extracted from tissues using 80% aqueous acetone buffered with 2.5 mmol/L sodium phosphate buffer (pH 7.8), and its levels were determined according to the method described by Porra et al. (1989). The presence of the transgene was confirmed by the PCR method, using Blend Taq polymerase (Toyobo, Osaka, Japan) and the primers 5′‐ATGAAGACACTTTCCAGCGC‐3′ and 5′‐TGGCAAATGCCTCTCAAGG‐3′, and the data for plants not carrying the transgene were omitted from the analysis.

### 2.8 DNA microarray‐based transcriptome analysis

Total RNA was extracted from 3‐ and 5‐day‐old rice suspension cells grown in the media supplemented with 0 or 0.3 mmol/L biuret using RNeasy Plant Mini Kit (Qiagen, Hilden, Germany). Two‐color microarray analysis with two biological replicates was performed using Agilent Rice Oligo DNA Microarray 4x44K slide (Agilent, Santa Clara, CA, USA) according to the manufacturer's instructions to estimate the ratio of transcript abundance between the treatments at each culture period. Cyanine 3 or cyanine 5 labeled antisense cRNA was synthesized from the total RNA sample using Agilent Quick Amp Labeling Kit and hybridized to the microarray slide at 65°C for 17 hr using Agilent Gene Expression Hybridization Kit. The slide was scanned using Agilent DNA microarray scanner. Data were extracted with Agilent Feature Extraction software. Differentially expressed genes (DEGs) were detected using an R software package limma (Ritche et al., 2015). Genes were considered to be differentially expressed if the *p*‐value was less than 0.005.

### 2.9 Functional classification of differentially expressed genes

Rice loci corresponding to the probes were confirmed using blastn searches against IRGSP‐1.0 genes_2020‐06‐03 that was downloaded from the Rice Annotation Project (RAP) database (https://rapdb.dna.affrc.go.jp). Probes whose sequence was not mapped on rice locus or mapped on multiple loci were omitted from the subsequent analysis. When there were several probes corresponding to a locus, and when there was a marked inconsistency in the expression data, such a gene was omitted from DEGs' lists.

Gene ontology (GO) terms associated with the rice locus were obtained through the PANTHER Classification System (Mi et al., 2019) using the RAP gene identifiers as queries. Kyoto Encyclopedia of Genes and Genomes (KEGG) pathway identifiers for rice genes (KEGG organism code: dosa) were obtained from the KEGG database (Kanehisa & Goto, 2000; https://www.genome.jp/kegg/). Overrepresentation of these annotations in DEGs was tested by fisher's exact test, and Benjamini‐Hochberg adjusted *p*‐values < 0.05 was set as a threshold. Transcription factor and transcriptional regulator genes were identified according to the classification in the Plant Transcription Factor Database version 3.0 (Pérez‐Rodríguez et al., 2010; http://plntfdb.bio.uni‐potsdam.de/v3.0/).

### 2.10 Accession

The sequenced data of *Rhizobium* sp. KaB01 *biuret hydrolase* has been submitted to the DDBJ/EMBL/GenBank databases under the accession number LC532383.

## 3 RESULTS

### 3.1 Biuret injury in hydroponically grown rice seedlings

**FIGURE 1:**
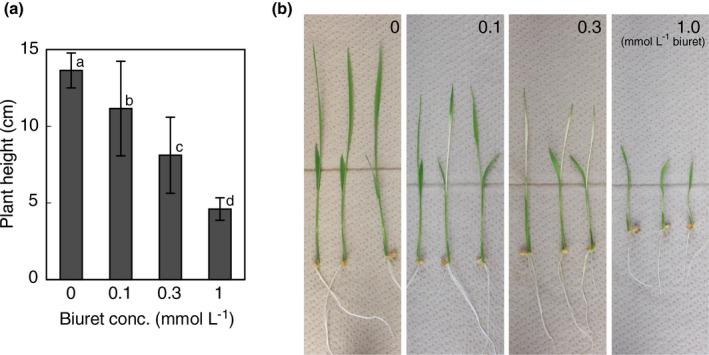
Symptoms of biuret toxicity in rice plants. Rice seedlings were hydroponically grown in a culture solution supplemented with 0, 0.1, 0.3, and 1.0 mmol/L biuret. (a) Plant heights of 7‐day‐old seedlings. The data represent means ± *SD* (*n* = 22–24). Different alphabets indicate significant differences between groups (*p* < 0.05, Tukey test). (b) Representative images of 7‐day‐old seedlings

We first investigated the biuret sensitivity of wild‐type rice plants. When wild‐type Nipponbare seeds were sown in culture solutions supplemented with varying levels of biuret (0, 0.1, 0.3, and 1.0 mmol/L biuret), germination was not inhibited, regardless of the biuret levels. The plant height of 7‐day‐old seedlings decreased with increasing concentrations of biuret (Figure 1a). Chlorosis was observed in some plants exposed to 0.1 mmol/L biuret and all plants exposed to 0.3 mmol/L biuret. It was most markedly observed in leaf blades of the emerging third leaf. In plants exposed to 1.0 mmol/L biuret, chlorosis was less prominent, probably because of the severe reduction in the growth (Figure 1b).

### 3.2 Biuret uptake by rice seedlings

Prior to the evaluation of biuret uptake by rice plants, we first examined biuret decomposition in culture solutions. Containers filled with 1 L of culture solution supplemented with 0.3 or 1.0 mmol/L biuret were placed in a growth chamber, and aliquots of culture solutions were collected from the containers at 0, 12, 24, 48, and 72 hr. The biuret concentrations were allowed to remain constant for up to 72 hr. This result indicated that biuret was not hydrolyzed in the culture solution within this period.

Then, we carried out 48‐hr uptake experiments using ^15^N‐labelled biuret. Biuret can be measured as a colored chelation complex with cupric ions or by HPLC analysis combined with UV‐detection, which we used for the determination of biuret concentrations in the culture solution. However, the sensitivity of the colorimetry process was too low to determine biuret concentrations in plant samples. Additionally, the peak of biuret could not be separated from UV‐absorbing metabolites of rice plants in our system.

When 19‐day‐old Nipponbare seedlings were allowed to take up ^15^N‐labeled biuret for 48 hr, biuret‐derived ^15^N was detected both in the shoots and roots of the seedlings (Table 1). This result indicated that biuret was taken up by rice roots and possibly translocated into rice shoots. The biuret‐derived ^15^N concentrations in the shoots and roots were equal to 4.5 and 1.9 µmol biuret g^−1^ dw, respectively. Then, based on the amount of biuret in whole seedlings, the uptake rate of biuret with an external supply of 0.3 mmol/L was calculated at 0.5 µmol/g root dw h^−1^.

**Table 1:** ^15^N‐Biuret uptake in rice plants. Nineteen‐day‐old Nipponbare seedlings were exposed to 0.3 mmol/L ^15^N‐biuret for 48 hr. Two seedlings were incubated in each of three 40‐mL vials, and plants in a vial were analyzed as one sample. Values are expressed as means ± *SD* (*n* = 3)

	Dry weight	Total‐N	Biuret‐derived N	
	(mg vial^−1^)	(mmol/g DW)	(µmol/g DW)	(µmol vial^−1^)
Shoots	124 ± 3.88	2.43 ± 0.09	13.5 ± 1.02	1.67 ± 0.18
Roots	23.8 ± 1.06	1.75 ± 0.01	5.83 ± 0.55	0.13 ± 0.01

### 3.3 Transgene expression and biuret decomposing activity in transgenic rice plants

To examine the effect of biuret hydrolysis in plant cells on the biuret tolerance of rice plants, we generated transgenic rice plants overexpressing *biuret hydrolase* that were cloned from *Rhizobium* sp. KaB01. The inserted *biuret hydrolase* encoded a protein consisting of 238 amino acid residues (Figure S2), whose amino acid sequence showed 92% similarity to a known biuret hydrolase of *R. leguminosarum* bv. *viciae* 3841 (WP_011654379.1; Cameron et al., 2011). In addition, purified recombinant maltose‐binding protein fusion proteins expressed in *Escherichia coli* showed the biuret decomposing activity (Figure S3). Signal peptides were not detected via the SignalP‐5.0 program (Almagro Armenteros et al., 2019), and the protein was predicted to be cytoplasmic localized, using the program PSORTb v.3.0 (Yu et al., 2010).

**FIGURE 2:**
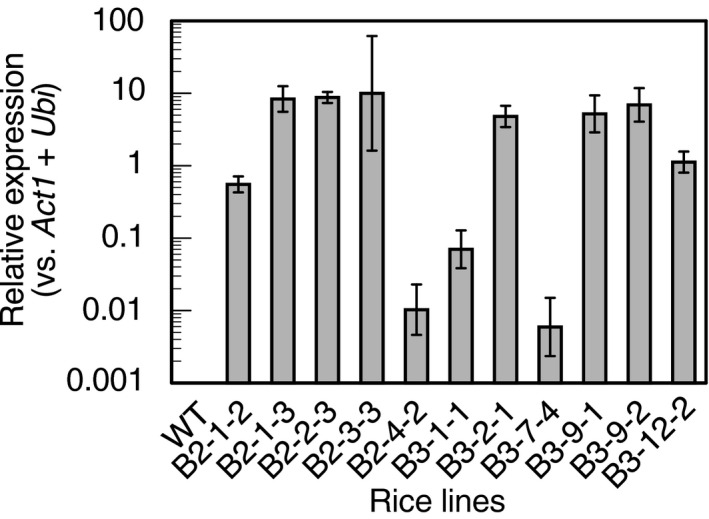
Relative expression levels of *biuret hydrolase* in transgenic rice plants. The expression in the youngest leaf at the vegetative growth stage was determined via real‐time PCR. *Ubiquitin* and *Actin1* were used as internal standards for normalization. The values are means of two technical replicates. Error bars indicate ranges of relative expression levels calculated from standard deviations of ∆Ct value. In names of transgenic lines, the combination of the first and second numbers indicates a callus derived from a single seed, and the third number indicates an individual plant regenerated from the callus

The *b* *iuret hydrolase* transgene was expressed in varying levels in regenerated T_0_ rice leaves, while its expression was not detected in wild‐type Nipponbare (Figure 2). Self‐pollinated progenies of two independent transgenic lines, B2‐3‐3 and B3‐9‐1, which showed high expression levels, were used for further examination.

**FIGURE 3:**
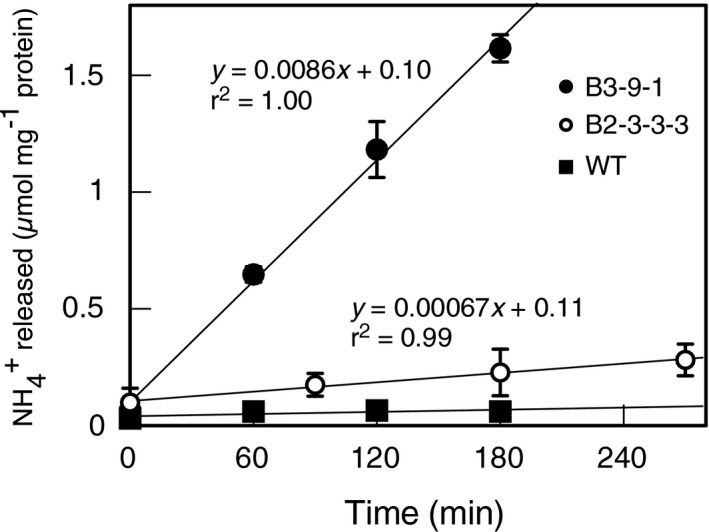
Biuret‐decomposing activities of crude extracts of rice plants. Crude extracts were prepared from shoots of individual 19‐day‐old wild‐type Nipponbare (solid boxes, *n* = 3) and transgenic B3‐9‐1 (solid circles, *n* = 2) plants and 16‐day‐old B2‐3‐3‐3 (open circles, *n* = 2). Extracts were incubated at 30ºC with 50 mmol/L sodium phosphate buffer (pH 8) and 3 mmol/L biuret. The reaction was stopped by adding 0.5 mol/L H_2_SO_4_. The amount of ammonia released from biuret was determined using indophenol blue. The data represent means ± *SD*

Crude extracts prepared from 19‐day‐old seedlings of B2‐3‐3‐3 (T_2_) or 16‐day‐old seedlings of B3‐9‐1 (T_1_) showed the biuret decomposing activity. In both lines, the amount of ammonia released from biuret increased linearly with time (Figure 3). The line B3‐9‐1 showed higher specific activity than B2‐3‐3‐3. The specific activities of extracts from the B3‐9‐1 and B2‐3‐3‐3 plants were 8.6 and 0.67 nmol/min mg^−1^ protein. The result was inconsistent with the similar transgene expression levels in these lines. It might be affected by the relationship between gene expression and protein expression. Wild‐type Nipponbare plants did not exhibit ammonia‐releasing activity (Figure 3). Extracts heated at 100ºC for 5 min were inactive. In addition, extracts prepared from a null segregant of B3‐9‐1 did not exhibit ammonia‐releasing activity. These results indicated that the transgenic rice lines were conferred with biuret decomposing ability by the exogenous *biuret hydrolase*.

### 3.4 Biuret tolerance in transgenic rice plants overexpressing *biuret hydrolase*

**FIGURE 4:**
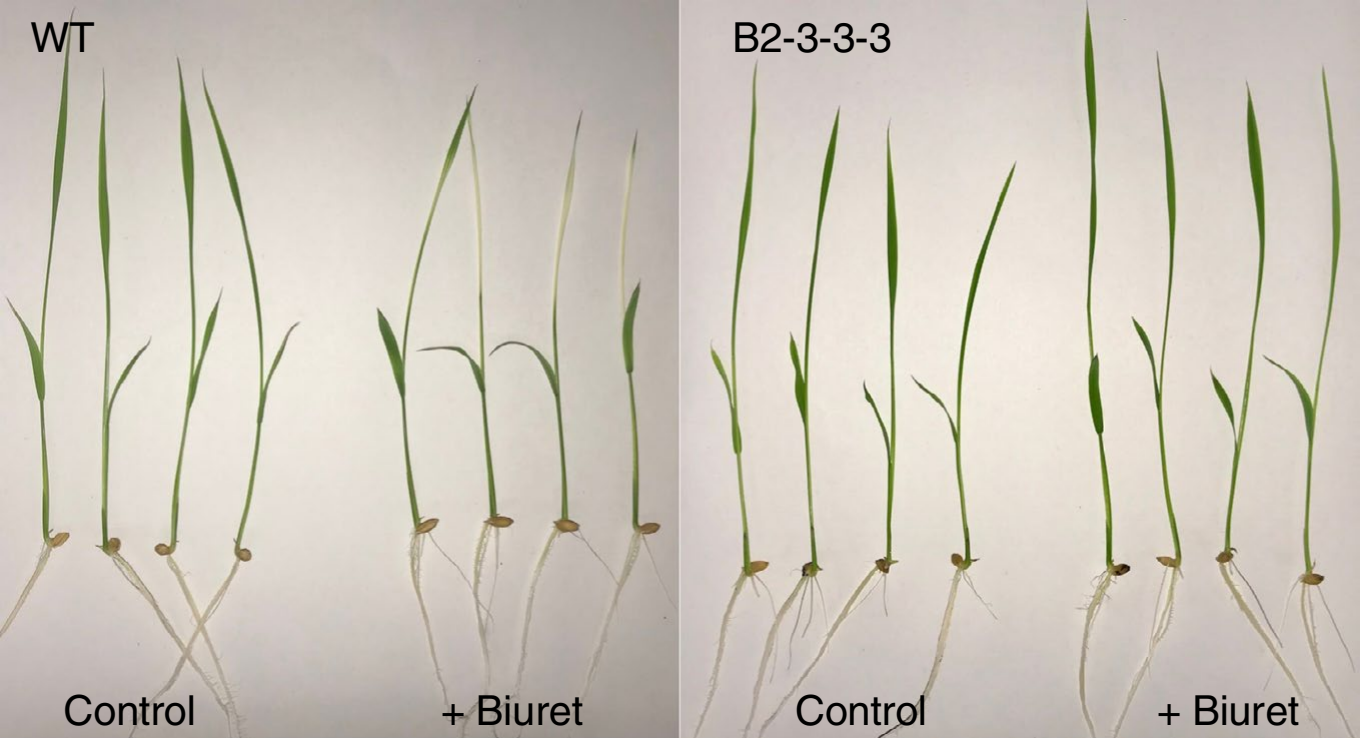
Biuret tolerance of a transgenic rice line overexpressing exogenous *biuret hydrolase*. Biuret treatment (0 and 0.3 mmol/L) was started two days after the sowing process. Photos were taken 9 days after sowing. Wild‐type rice plants (left panel) and transgenic rice plants (line B2‐3‐3‐3, right panel) grown without and with biuret

**FIGURE 5:**
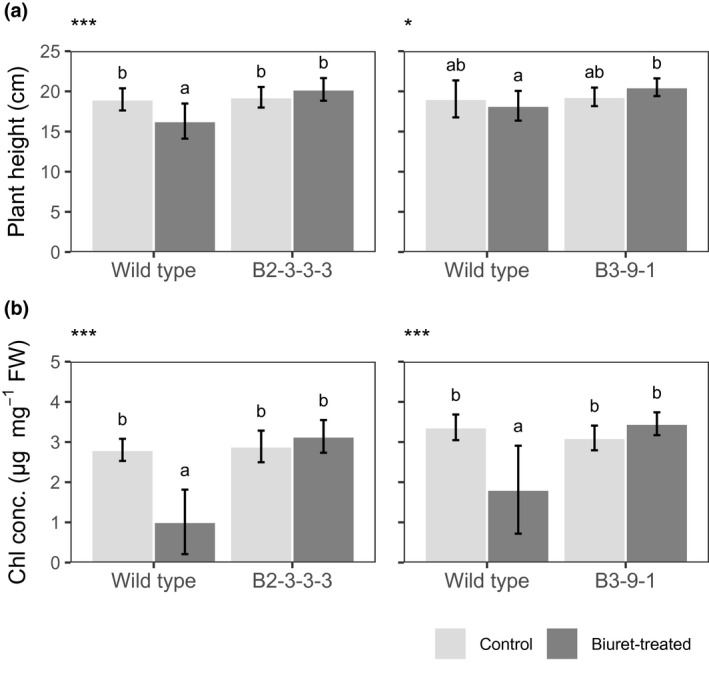
Plant height and chlorophyll content of wild‐type (WT) and *biuret hydrolase*‐overexpressing rice plants under the effect of biuret toxicity. (a) Plant height, and (b) chlorophyll content in third leaf. Biuret treatment (0.3 mmol/L) was started two days after sowing, and nine plants were harvested. Two transgenic lines, B2‐3‐3‐3 and B3‐9‐1, were grown in separate experiments. Light gray boxes indicate control plants, and dark gray boxes indicate biuret‐treated plants. Values are expressed means ± *SD* (*n* = 12–16). Asterisks on the top of panels indicate the significance of the interaction between the line and treatment (Two‐way ANOVA, *p < 0.05; ***p <0 .001). Different alphabets indicate significant differences among groups (*p* < 0.05, Tukey's test)

Rice plants overexpressing biuret hydrolase showed improved tolerance to biuret (Figure 4). The plant height of 9‐day‐old wild‐type rice seedlings was reduced or remained unchanged with the addition of 0.3 mmol/L biuret. It was slightly increased in two transgenic lines, although the difference was not statically significant (Figure 5a). Similar results were also obtained for the chlorophyll content observed in leaf blades of the third leaf. The chlorophyll content in wild‐type plants decreased significantly in the presence of excessive biuret levels but slightly and not significantly increased in B3‐9‐1 and B2‐3‐3‐3 plants (Figure 5b). These results clearly showed that rice plants overexpressing *biuret hydrolase* gained tolerance to biuret toxicity through the decomposition of biuret in plants.

### 3.5 Biuret uptake in transgenic rice plants

The slight increment in plant heights of biuret‐treated transgenic lines suggested that biuret was utilized as an additional N source. Therefore, we also determined biuret uptake in *biuret hydrolase*‐overexpressing lines. The concentration of biuret‐derived N was 2.6 and 5.6 times higher in two transgenic lines, B2‐3‐3‐11 (T_2_) and B3‐9‐1‐5 (T_2_), as compared to that in wild‐type plants (Table 2). This suggested that biuret was decomposed in the roots and that ^15^N derived from biuret was built into more mobile compounds, such as amino acids, and transported into shoots. Taken together, *biuret hydrolase*‐overexpressing plants seemingly used biuret as an extra N source.

**Table 2:** Biuret‐derived ^15^N in shoots of *biuret hydrolase*‐overexpressing rice plants. Nineteen‐day‐old seedlings were exposed to 0.3 mmol/L^15^N‐biuret for 48 hr. Two seedlings were incubated in each of 40‐mL vials, Values are expressed means ± *SD* (WT: *n* = 2, B2‐3‐3‐11 and B3‐9‐1‐5: *n* = 3)

Line	Dry weight	Total‐N	Biuret‐derived N	
	(mg vial^−1^)	(mmol/g DW)	(µmol/g DW)	(µmol vial^−1^)
WT	109^b^ ± 11.2	2.30^a^ ± 0.13	16.4^a^ ± 1.76	1.80^a^ ± 0.38
B2‐3‐3‐11	76.2^a^ ± 6.67	2.35^a^ ± 0.21	42.3^b^ ± 5.39	3.20^b^ ± 0.13
B3‐9‐1‐5	77.6^a^ ± 5.79	2.42^a^ ± 0.12	91.4^c^ ± 1.98	7.09^c^ ± 0.59

Note

Different alphabets indicate significant differences between rice lines (*p* < 0.05, Tukey's test).

### 3.6 Transcriptome analysis of rice suspension cells under biuret toxicity

**FIGURE 6:**
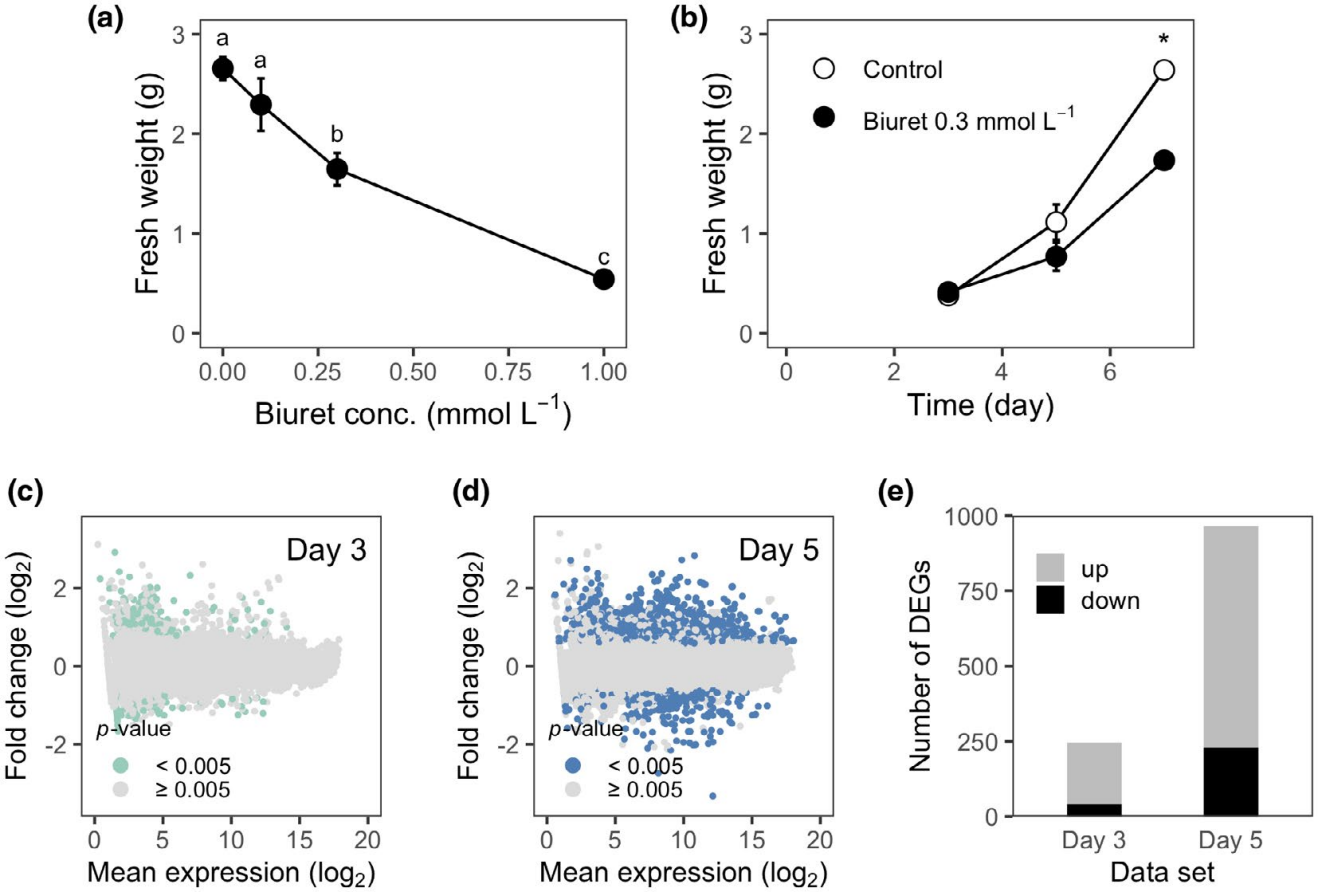
Microarray‐based transcriptome analysis in rice suspension cells under biuret toxicity. (a) Biuret toxicity in rice suspension cells. Cells were subcultured into a culture medium supplemented with 0, 0.1, 0.3, and 1.0 mmol/L biuret. Fresh cell weight per culture flask was measured seven days after subcloning. Values are expressed means ± *SD* (*n* = 3). Different alphabets indicate significant differences among groups (*p* < 0.05, Tukey's test). (b) Changes in the fresh cell weight over time. Rice cells were subcultured into the culture medium supplemented with 0 and 0.3 mmol/L biuret on day 0. The cells on day three and day 5 were used for subsequent microarray analyses. White circles indicate the control cells, and black circles indicate biuret‐treated cells. Values are expressed means ± *SD* (*n* = 2). An asterisk indicates a significant difference between the treatments (*p* < 0.05, *t* test). (c) Relationship between the normalized average gene expression levels and fold changes of gene expression of the 3‐day‐old biuret‐treated cells to the control cells. Each symbol denotes each microarray spot. Values are geometrical means of two arrays (*n* = 2). (d) Relationship between the normalized average gene expression levels and fold changes of gene expression of the 5‐day‐old biuret‐treated cells to the control cells. Each symbol denotes each microarray spot. Eleven points with high non‐significant fold‐change are omitted from the figure. Values are geometrical means of two arrays (*n* = 2). (e) Numbers of differentially expressed genes

Lastly, gene expression changes in response to biuret toxicity were examined in rice suspension cells. When rice suspension cells were subcultured into the culture medium supplemented with 0–1 mmol/L biuret, the fresh weight of 7‐day‐old cells decreased with increasing biuret concentration in the culture medium (Figure 6a). The reduction in growth was significant at biuret concentrations above 0.3 mmol/L. Rice cells hardly proliferated under 1 mmol/L biuret toxicity. Figure 6b shows representative data for the changes in the fresh weights over time for the control and 0.3 mmol/L biuret‐treated cells. Cells kept growing until seven days after subculturing. The difference between the treatments in cell fresh weight was not significant until day 5 but was significant by day 7 (Figure 6b).

The cells on days 3 and 5 (Figure 6b) were used for the subsequent microarray‐based transcriptome analysis in which gene expression was compared between the biuret treatments at each culture period. The gene expression levels on 436 and 1,743 microarray spots were significantly different between the treatments in cells on days 3 and 5, respectively (Figure 6c,d). As genes, 246 (204 up‐ and 42 down‐regulated) and 966 (736 up‐ and 230‐down‐regulated) loci of the total 22,794 loci were considered as differentially expressed genes (DEGs) for day 3 and day 5 datasets (Figure 6e, Dataset S1 and DatasetS2). Forty‐seven of these genes were differentially expressed in both the culture period.

Then, we performed GO enrichment analysis and KEGG pathway enrichment analysis to know the biological significance of the DEGs. One‐hundred‐forty‐five DEGs on day 3 and 704 DEGs on day 5 were annotated to one or more GO terms. Similarly, 23 DEGs on day 3 and 165 DEGs on day 5 were mapped onto KEGG pathways. Any significant (adjusted‐*p* < 0.05) overrepresentation of GO terms or KEGG pathways was not detected in DEGs on day 3.

**FIGURE 7:**
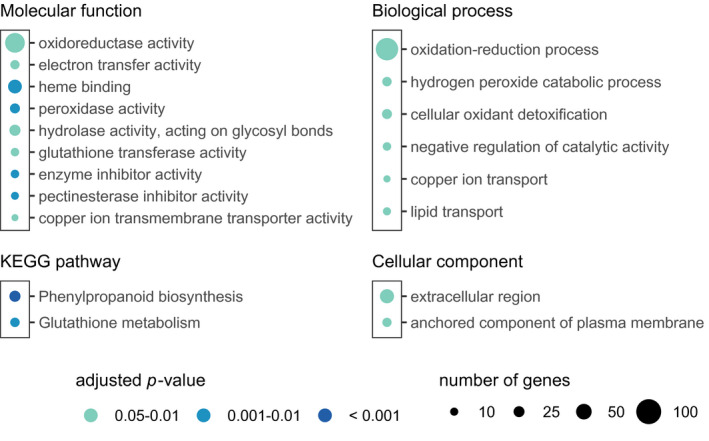
GO term and KEGG pathway enrichment analysis for differentially expressed genes in 5‐day‐old rice suspension cells under biuret toxicity. The overrepresentation was tested by Fisher's exact test, and terms with an adjusted *p*‐value below 0.05 are listed

Seventeen GO terms and three KEGG pathways were overrepresented in DEGs on day 5 (Figure 7, Figure S4). The overrepresented terms indicated that metabolic reactions involved in cellular redox regulation were active in the biuret‐treated cells and thus suggested the accumulation of reactive oxygen species (ROS) in the cells. Terms such as “Oxidation‐reduction process” (GO: 0055114), “hydrogen peroxide catabolic process” (GO: 0042744), “cellular oxidant detoxification” (GO: 0098869), “oxidoreductase activity” (GO: 0016491), “heme binding” (GO: 002037), “peroxidase activity” (GO: 0004601), and “electron transfer activity” (GO: 0009055) were directly related to cellular redox conditions. Two other terms, “copper ion transport” (GO: 0006825) and “copper ion transmembrane transporter activity” (GO: 0005375), were also relevant to redox regulations, for copper is an essential redox‐active transition metal. As well‐known, biuret forms chelation complexes with copper ions in a strongly alkaline solution; however, it is uncertain whether biuret directly interacts with copper ions in rice cells.

The enrichment analyses also indicated active adaptive responses those similar to responses to a broad range of abiotic and biotic stresses. “Phenylpropanoid biosynthesis” (dosa00940) pathway starts with phenylalanine and ends with phenolic compounds, including flavonoids and lignin, has been known to be activated under stresses (Dixon & Paiva, 1995). “Glutathione metabolism pathway” (dosa009480) and “glutathione transferase activity” (GO: 0004364) are essential for cellular redox control (Dixon et al., 2002). “Pectinesterase inhibitor activity” (GO: 0046910) and some genes categorized under “hydrolase activity, acting on glycosyl bonds” (GO: 0016798) relates stiffening or loosening of cell walls (Hong et al., 2010; Sharma et al., 2013). “Lipid transport” (GO: 0006869) is also involved in stress adaptation (Guo et al., 2013; Zhao et al., 2020).

Besides, approximately 8% of DEGs consisted of transcription factor genes and other transcription regulator genes (Dataset S1 and Dataset S2). Only two genes, one *MYB* gene, Os08g0151300, and one *TCP* gene, Os02g0747400, were shared between the culture periods. Both of them were up‐regulated. Os08g0151300 (*CEF1/OsMYB103L*) was reported to regulate cellulose synthesis (Yang et al., 2014).

Transcription factor genes in DEGs included genes known to be regulating stress responses besides genes known to be regulating growth and development. In DEGs on day 3, there were five stress‐responsive genes: two *AP2‐EREBP* genes Os09g0522000 and Os09g0522200 (*OsDREB1B* and *OsDREB1A*; Dubouzet et al., 2003), a *bHLH* gene Os04g0301500 (*OsbHLH6*; Meng et al., 2020), a *C3H* gene Os05g0128200 (*OsTZ5*; Selvaraj et al., 2020), and a *NAC* gene Os08g0436700 (*ONAC063*; Yokotani et al., 2009). These genes could be induced by cold, drought, salt, or fungal infection. Additionally, we investigated GO terms associated with DEGs and started with response to or defense response to and found water deprivation, salt, cold, wounding, fungus, bacterium, and oomycetes after these words (Table S1).

Taken together, results of microarray analysis suggested that ROS accumulated in 5‐day‐old rice cells under 0.3 mmol/L biuret toxicity to cause oxidative stress. The molecules involved in response to biuret stress had some similarities with those for other environmental stresses. Furthermore, two overrepresented terms in the cell component category suggested metabolisms in the apoplast were active.

## 4 DISCUSSION

Although biuret toxicity in crops is a well‐known issue, little is known about the physiology underlying biuret injury. Here, we analyzed for the first time biuret uptake in rice plants quantitatively using ^15^N‐labelled biuret and revealed that a considerable amount of biuret was taken up by wild‐type rice (Table 1). As wild‐type rice plants did not show any biuret decomposing activity (Figure 3), biuret‐derived ^15^N is considered as biuret in plants. Therefore, the shoot biuret concentration is approximately equal to 0.4 mmol/L when it is expressed on the basis of the tissue water content. The concentration of biuret was higher in shoots than in roots, which indicates that the amount of biuret retained in roots was small, and that biuret was accumulated in shoots through the transpiration stream. As biuret is a small polar molecule without lipophilic parts, cellular membranes may be slightly permeable to biuret. The rate of biuret uptake was calculated from the ^15^N content in whole seedlings, and was found to be equivalent to 0.5 µmol/g root dw h^−1^. For comparisons, the rate of urea influx into roots, which is largely mediated by channels and a high‐affinity transporter, were about 20 µmol/g root dw h^−1^ in *Arabidopsis thaliana* (Kojima et al., 2007) and about 6 µmol/g root dw h^−1^ in rice (Wang et al., 2012), when 0.3 mmol/L urea was supplied as a sole N source. The observed uptake rate of biuret was one to two orders of magnitudes lower than that of urea. Similarly, the permeability of biuret could not be detected at 10ºC in mouse erythrocytes that were permeable to urea (Zhao et al., 2007). Biuret could possibly move across membranes via simple diffusion. To evaluate biuret accumulation over a prolonged period, we need to develop a method to detect biuret directly. We are currently modifying HPLC methods, to separate biuret from other UV‐absorbing compounds in plants.

The overexpression of bacterial *biuret hydrolase* conferred biuret tolerance to rice plants (Figure 4, Figure 5). Conversely, biuret was seemingly not metabolized, or very slowly metabolized in wild‐type rice plants. This is consistent with our enzyme assay results, obtained using leaf crude extracts (Figure 3), and with the previous report on biuret in orange leaves, in which biuret was detected by the eight months after foliar application by a qualitative analysis (Impey & Jones, 1960). The lack of an efficient decomposition pathway is probably responsible for biuret accumulation and toxicity in rice plants. Besides, biuret tolerance conferred by the *biuret hydrolase* suggested that an injury in rice plants occurred because of the direct effects of biuret within plants, but not from the indirect effects of biuret outside roots.

Additionally, our results on the biuret injury in wild‐type rice plants and rice suspension cells gave some indications of mechanisms underlying biuret toxicity. In wild‐type rice seedlings, a biuret concentration of 0.1 mmol/L and above in the culture solution caused a significant reduction in the growth (Figure 1). It was roughly consistent with the toxic concentration of biuret reported for hydroponically grown naked barley (Funabiki et al., 1956) and pot cultured young citrus and avocado plants (Haas & Brusca, 1954). The rather high dose, together with the significant accumulation of biuret in rice shoots, suggests that biuret is moderately toxic and that biuret might have a weak affinity with its target.

The occurrence of leaf chlorosis was observed, along with growth inhibition, in biuret‐injured rice seedlings (Figure 1). The colorless appearance of elongating young leaves indicates that excessive biuret impaired chloroplast development. Closely similar chlorosis was often observed in rice seedlings exposed to cold stress (Yoshida et al., 1996). It also occurs in seedlings suddenly exposed to direct sunlight after germination in the darkness during preparation for rice seedlings for machine transplanting. It has been shown that cold stress especially impairs the establishment of the plastid genetic system during chloroplast development in rice seedlings (Kusumi et al., 2011). Biuret might trigger similar downstream cellular responses.

The chlorosis and reduced growth in rice seedlings are possibly not in a causal relationship. They may be parallel since biuret causes growth inhibition even in heterotrophic suspension cells of rice (Figure 6a). It is unlikely that decreased photosynthetic activity caused reduced growth in suspension cells.

ROS seem to be involved in the upstream of such biuret injury (Figure 7). As well‐known, excess ROS cause severe cellular damages. Additionally, ROS under proper regulation are necessary for cellular and systemic signal transduction in plants (Fichman & Mittler, 2020; Miller et al., 2009). Plasma membrane‐localized NADPH oxidases, or respiratory burst homolog (Rboh), produce ROS. The produced ROS first accumulate in the apoplast, and then go into the cytosol or diffuse toward neighboring cells for signaling (Fichman & Mittler, 2020). Apoplastic ROS are also used for cell wall stiffening and loosening (Kärkönen & Kuchitsu, 2015). In our GO enrichment analysis, the term “peroxidase activity” is overrepresented (Figure 7, Figure S4). This suggests the enhanced activity of peroxidases under biuret toxicity. Cell wall‐localized peroxidases could act in cross‐linking phenolic compounds using H_2_O_2_ as an oxidant and thus tightening cell walls (Cosio & Dunand, 2009). At the same time, they could act in the cleavage of cell wall polysaccharides through the production of ·OH and thus loosening cell walls (Liszkay et al., 2003). Moreover, two overrepresented terms, “Pectinesterase inhibitor activity” and “hydrolase activity, acting on glycosyl bonds” were also related to cell wall polysaccharide modification. Therefore, it is likely that modifications of cell walls that are often found in plants under abiotic stresses (Tenhaken, 2015) also occur under biuret toxicity. The rigidity of cell walls restricts cell elongation. Our results do not show whether the cell wall rigidity changes or not. Enzyme activity and metabolites should also be examined. However, it may be a cause of retarded growth of rice plants under biuret toxicity.

The gene expression change of rice suspension cells under biuret toxicity showed some similarities with various environmental stresses and plant hormones (Figure 7, Table S1). Overall responses for stress in plants result from a combination of signal transductions through pathways specific to the stress and common to other stresses (Knight & Knight, 2001; Sewelam et al., 2016). The pathway of the response to biuret excess in rice cells also appears to be complicated. Biuret is commercially used as non‐protein nitrogen to feed ruminants; that is, there is no known toxicity to animals. Therefore, we have postulated that biuret would inhibit some plant‐specific metabolic reactions. However, plant‐specific elaborated protection systems for stresses might be the cause of plant‐specific toxicity of biuret. The site of action of biuret is still unknown. Further research should be focused on this point.

In conclusion, the findings reported here clearly demonstrate that it is possible to confer biuret detoxification ability on rice plants by introducing the microbial *biuret hydrolase* (Figure 4, Figure 5). Moreover, rice plants overexpressing *biuret hydrolase* utilize ammonium‐N produced by the hydrolysis of biuret in plant cells as an additional N source (Table 2). In soil, the decomposition rate, or the mineralization rate, of biuret is slower than that of urea (Ogata & Funabiki, 1956; Sahrawat, 1981). Taken together, when biuret is applied as a N fertilizer to the transgenic rice lines that were generated here, the fertilizer use efficiency would possibly be improved compared with that of urea fertilization. We are currently working on soil‐culture experiments to evaluate the effect of biuret as a N fertilizer.

## AUTHOR CONTRIBUTIONS

K.O. and T.M. conceived and designed the study and supervised the experiments. A.U. performed the experiments on intact plants. Y.M. isolated the soil bacterium. M.N. performed the experiments on suspension cells. K.O. contributed to the preliminary experiments, analyzed microarray data, and wrote the manuscript. All authors contributed to the writing of the manuscript.

## Supporting information

Supinfo S1

Supinfo S2

Supplemental Data S1

Supplemental Data S2

## References

[pld3290-bib-0001] Achor, D. S. , & Albrig, L. G. (2005). Biuret toxicity symptoms in citrus leaves mimics cell senescence rather than nutritional deficiency chlorosis. Journal of the American Society for Horticultural Science, 130, 667–673. 10.21273/JASHS.130.5.667

[pld3290-bib-0002] Almagro Armenteros, J. J. , Tsirigos, K. D. , Sønderby, C. K. , Petersen, T. N. , Winther, O. , Brunak, S. , von Heijne, G. , & Nielsen, H. (2019). SignalP 5.0 improves signal peptide predictions using deep neural networks. Nature Biotechnology, 37, 420–423. 10.1038/s41587-019-0036-z 30778233

[pld3290-bib-0003] Aukema, K. G. , Tassoulas, L. J. , Robinson, S. L. , Konopatski, J. F. , Bygd, M. D. , & Wackett, L. P. (2020). Cyanuric acid biodegradation via biuret: Physiology, taxonomy, and geospatial distribution. Applied and Environmental Microbiology, 86, e01964–e2019. 31676480 10.1128/AEM.01964-19 PMC6952224

[pld3290-bib-0004] Baba, A. , Hasezawa, S. , & Syōno, K. (1986). Cultivation of rice protoplasts and their transformation mediated by *Agrobacterium* spheroplasts. Plant and Cell Physiology, 27, 463–471.

[pld3290-bib-0005] Cameron, S. M. , Durchschein, K. , Richman, J. E. , Sadowsky, M. J. , & Wackett, L. P. (2011). New family of biuret hydrolases involved in *s*‐Triazine ring metabolism. ACS Catalysis, 1, 1075–1082. 10.1021/cs200295n PMC3166513 21897878

[pld3290-bib-0007] Cheng, G. , Shapir, N. , Sadowsky, M. J. , & Wackett, L. P. (2005). Allophanate hydrolase, not urease, functions in bacterial cyanuric acid metabolism. Applied and Environmental Microbiology, 71, 4437–4445. 10.1128/AEM.71.8.4437-4445.2005 16085834 PMC1183272

[pld3290-bib-0008] Cosio, C. , & Dunand, C. (2009). Specific functions of individual class III peroxidase genes. Journal of Experimental Botany, 60, 391–408. 10.1093/jxb/ern318 19088338

[pld3290-bib-0009] Dixon, D. P. , Lapthorn, A. , & Edwards, R. (2002). Plant glutathione transferases. Genome Biology, 3, 1–10. REVIEWS3004. 10.1186/gb-2002-3-3-reviews3004 PMC139027 11897031

[pld3290-bib-0010] Dixon, R. A. , & Paiva, N. L. (1995). Stress‐induced phenylpropanoid metabolism. The Plant Cell, 7, 1085–1097. 12242399 10.1105/tpc.7.7.1085 PMC160915

[pld3290-bib-0011] Dubouzet, J. G. , Sakuma, Y. , Ito, Y. , Kasuga, M. , Dubouzet, E. G. , Miura, S. , Seki, M. , Shinozaki, K. , & Yamaguchi‐Shinozaki, K. (2003). OsDREB genes in rice, *Oryza sativa* L., encode transcription activators that function in drought‐, high‐salt‐ and cold‐responsive gene expression. The Plant Journal, 33, 751–763. 12609047 10.1046/j.1365-313x.2003.01661.x

[pld3290-bib-0012] Esquirol, L. , Peat, T. S. , Sugrue, E. , Balotra, S. , Rottet, S. , Warden, A. C. , Wilding, M. , Hartley, C. J. , Jackson, C. J. , Newman, J. , & Scott, C. (2020). Bacterial catabolism of s‐triazine herbicides: Biochemistry, evolution and application. Advances in Microbial Physiology, 76, 129–186. 32408946 10.1016/bs.ampbs.2020.01.004

[pld3290-bib-0013] Esquirol, L. , Peat, T. S. , Wilding, M. , Lucent, D. , French, N. G. , Hartley, C. J. , Newman, J. , & Scott, C. (2018). Structural and biochemical characterization of the biuret hydrolase (BiuH) from the cyanuric acid catabolism pathway of *Rhizobium leguminasorum* bv. *viciae* 3841. PLoS One, 13, e0192736. 29425231 10.1371/journal.pone.0192736 PMC5806882

[pld3290-bib-0014] Fichman, Y. , & Mittler, R. (2020). Rapid systemic signaling during abiotic and biotic stresses: Is the ROS wave master of all trades? The Plant Journal, 102, 887–896. 10.1111/tpj.14685 31943489

[pld3290-bib-0015] Funabiki, S. , Ogata, T. , & Sakamoto, T. (1956). Studies on biuret from agricultural standpoint: I. Influences on plant growth. The Scientific Reports of the Matsuyama Agricultural College, 12, 1–14.

[pld3290-bib-0016] Guo, L. , Yang, H. , Zhang, X. , & Yang, S. (2013). Lipid transfer protein 3 as a target of MYB96 mediates freezing and drought stress in Arabidopsis. Journal of Experimental Botany, 64, 1755–1767. 10.1093/jxb/ert040 23404903 PMC3617838

[pld3290-bib-0017] Haas, A. R. C. , & Brusca, J. N. (1954). Biuret, toxic form of nitrogen: Soluble nitrogen compounds are not of equal value as fertilizers as shown by tests with citrus and avocado. California Agriculture, 8, 7–11.

[pld3290-bib-0018] Hewitt, E. J. (1966). The composition of the nutrient solution In Hewitt E. J. (Ed.), Sand and water culture methods used in the study of plant nutrition (p. 190). Commonwealth Agricultural Bureaux.

[pld3290-bib-0019] Hong, M. J. , Kim, D. Y. , Lee, T. G. , Jeon, W. B. , & Seo, Y. W. (2010). Functional characterization of pectin methylesterase inhibitor (PMEI) in wheat. Genes & Genetic Systems, 85, 97–106. 10.1266/ggs.85.97 20558896

[pld3290-bib-0020] Impey, R. L. , & Jones, W. W. (1960). Effects of biuret on nitrogen status of Washington navel and Valencia orange leaves. American Society for Horticultural Science, 76, 186–192.

[pld3290-bib-0021] Jensen, H. L. , & Schrøder, M. (1965). Urea and biuret as nitrogen sources for *Rhizobium* Spp. Journal of Applied Bacteriology, 28, 473–478. 5852362 10.1111/j.1365-2672.1965.tb02178.x

[pld3290-bib-0022] Jones, W. W. (1954). Biuret toxicity of urea foliage sprays on citrus. Science, 120, 499–500. 10.1126/science.120.3117.499 13195684

[pld3290-bib-0023] Kanehisa, M. , & Goto, S. (2000). KEGG: Kyoto encyclopedia of genes and genomes. Nucleic Acids Research, 28, 27–30. 10.1093/nar/28.1.27 10592173 PMC102409

[pld3290-bib-0024] Kärkönen, A. , & Kuchitsu, K. (2015). Reactive oxygen species in cell wall metabolism and development in plants. Phytochemistry, 112, 22–32. 10.1016/j.phytochem.2014.09.016 25446232

[pld3290-bib-0025] Knight, H. , & Knight, M. R. (2001). Abiotic stress signalling pathways: Specificity and cross‐talk. Trends in Plant Science, 6, 262–267. 10.1016/S1360-1385(01)01946-X 11378468

[pld3290-bib-0026] Kojima, S. , Bohner, A. , Gassert, B. , Yuan, L. , & von Wirén, N. (2007). AtDUR3 represents the major transporter for high‐affinity urea transport across the plasma membrane of nitrogen‐deficient Arabidopsis roots. The Plant Journal, 52, 30–40. 10.1111/j.1365-313X.2007.03223.x 17672841

[pld3290-bib-0027] Kusumi, K. , Sakata, C. , Nakamura, T. , Kawasaki, S. , Yoshimura, A. , & Iba, K. (2011). A plastid protein NUS1 is essential for build‐up of the genetic system for early chloroplast development under cold stress conditions. The Plant Journal, 68, 1039–1050. 10.1111/j.1365-313X.2011.04755.x 21981410

[pld3290-bib-0028] Liszkay, A. , Ken, B. , & Schopfer, P. (2003). Evidence for the involvement of cell wall peroxidase in the generation of hydroxyl radicals mediating extension growth. Planta, 217, 658–667. 10.1007/s00425-003-1028-1 12739149

[pld3290-bib-0029] Martinez, B. , Tomkins, J. , Wackett, L. P. , Wing, R. , & Sadowsky, M. J. (2001). Complete nucleotide sequence and organization of the Atrazine catabolic plasmid pADP‐1 from *Pseudomonas*sp. Strain ADP. Journal of Bacteriology, 183, 5684–5697. 11544232 10.1128/JB.183.19.5684-5697.2001 PMC95461

[pld3290-bib-0030] Meng, F. , Yang, C. , Cao, J. , Chen, H. , Pang, J. , Zhao, Q. , Wang, Z. , Qing, F. Z. , & Liu, J. (2020). A bHLH transcription activator regulates defense signaling by nucleo‐cytosolic trafficking in rice. Journal of Integrative Plant Biology, 62, 1552–1573. 10.1111/jipb.12922 32129570

[pld3290-bib-0031] Mi, H. , Muruganujan, A. , Ebert, D. , Huang, X. , & Thomas, P. D. (2019). PANTHER version 14: More genomes, a new PANTHER GO‐slim and improvements in enrichment analysis tools. Nucleic Acids Research, 47, D419–D426. 30407594 10.1093/nar/gky1038 PMC6323939

[pld3290-bib-0032] Mikkelsen, R. L. (1990). Biuret in Urea Fertilizer. Fertilizer Research, 26, 311–318. 10.1007/BF01048769

[pld3290-bib-0033] Miller, G. , Schlauch, K. , Tam, R. , Cortes, D. , Torres, M. A. , Shulaev, V. , Dangl, J. L. , & Mittler, R. (2009). The plant NADPH oxidase RBOHD mediates rapid systemic signaling in response to diverse stimuli. Science Signaling, 2, ra45 10.1126/scisignal.2000448 19690331

[pld3290-bib-0034] Nakagawa, T. , Suzuki, T. , Murata, S. , Nakamura, S. , Hino, T. , Maeo, K. , Tabata, R. , Kawai, T. , Tanaka, K. , Niwa, Y. , Watanabe, Y. , Nakamura, K. , Kimura, T. , & Ishiguro, S. (2007). Improved Gateway binary vectors: High‐performance vectors for creation of fusion constructs in transgenic analysis of plants. Bioscience, Biotechnology, and Biochemistry, 71, 2095–2100. 10.1271/bbb.70216 17690442

[pld3290-bib-0035] Ogata, T. , & Funabiki, S. (1956). Studies on biuret from agricultural standpoint: II. Stability of biuret in soil. The Scientific Reports of the Matsuyama Agricultural College, 12, 15–22.

[pld3290-bib-0036] Ogata, T. , & Yamamoto, M. (1959). Effects of biuret on the metabolism of germinating plant. I. Japanese Journal of Soil Science and Plant Nutrition, 29, 549–555. (in Japanese).

[pld3290-bib-0037] Pérez‐Rodríguez, P. , Riaño‐Pachón, D. M. , Corrêa, L. G. , Rensing, S. A. , Kersten, B. , & Mueller‐Roeber, B. (2010). PlnTFDB: Updated content and new features of the plant transcription factor database. Nucleic Acids Research, 38, D822–D827. 10.1093/nar/gkp805 19858103 PMC2808933

[pld3290-bib-0038] Porra, R. J. , Thompson, W. A. , & Kriedemann, P. E. (1989). Determination of accurate extinction coefficients and simultaneous equations for assaying chlorophylls *a* and *b* extracted with four different solvents: Verification of the concentration of chlorophyll standards by atomic absorption spectroscopy. Biochimica Et Biophysica Acta (BBA) ‐ Bioenergetics, 975, 384–394. 10.1016/S0005-2728(89)80347-0

[pld3290-bib-0039] Ritchie, M. E. , Phipson, B. , Wu, D. , Hu, Y. , Law, C. W. , Shi, W. , & Smyth, G. K. (2015). limma powers differential expression analyses for RNA‐sequencing and microarray studies. Nucleic Acids Research, 43, e47 10.1093/nar/gkv007 25605792 PMC4402510

[pld3290-bib-0040] Robinson, S. L. , Badalamenti, J. P. , Dodge, A. G. , Tassoulas, L. J. , & Wackett, L. P. (2018). Microbial biodegradation of biuret: Defining biuret hydrolases within the isochorismatase superfamily. Environmental Microbiology, 20, 2099–2111. 10.1111/1462-2920.14094 29528550

[pld3290-bib-0041] Sahrawat, K. L. (1981). Mineralization of biuret nitrogen in soil. Plant and Soil, 62, 46–471. 10.1007/BF02374144

[pld3290-bib-0042] Sanford, W. G. , Gowing, D. P. , Young, H. Y. , & Leeper, R. W. (1954). Toxicity to pineapple plants of biuret found in urea fertilizers from different sources. Sicence, 120, 349–350. 10.1126/science.120.3113.349 17753560

[pld3290-bib-0043] Selvaraj, M. G. , Jan, A. , Ishizaki, T. , Valencia, M. , Dedicova, B. , Maruyama, K. , Ogata, T. , Todaka, D. , Yamaguchi‐Shinozaki, K. , Nakashima, K. , & Ishitani, M. (2020). Expression of the CCCH‐tandem zinc finger protein gene *OsTZF5* under a stress‐inducible promoter mitigates the effect of drought stress on rice grain yield under field conditions. Plant Biotechnology Journal, 18, 1711–1721. 31930666 10.1111/pbi.13334 PMC7336284

[pld3290-bib-0044] Sewelam, N. , Kazan, K. , & Schenk, P. M. (2016). Global plant stress signaling: Reactive oxygen species at the cross‐road. Frontiers in Plant Science, 7, 187 10.3389/fpls.2016.00187 26941757 PMC4763064

[pld3290-bib-0045] Sharma, R. , Cao, P. , Jung, K. H. , Sharma, M. K. , & Ronald, P. C. (2013). Construction of a rice glycoside hydrolase phylogenomic database and identification of targets for biofuel research. Frontiers in Plant Science, 4, 330 10.3389/fpls.2013.00330 23986771 PMC3752443

[pld3290-bib-0046] Tanahashi, T. , Honda, M. , Takahashi, K. , & Yano, H. (2003). Analysis of chlorosis caused by resin‐coated urea used in nursery bed for paddy‐rice. Japanese Journal of Soil Science and Plant Nutrition, 74, 219–222. (in Japanese).

[pld3290-bib-0047] Tenhaken, R. (2015). Cell wall remodeling under abiotic stress. Frontiers in Plant Science, 5, 771 10.3389/fpls.2014.00771 25709610 PMC4285730

[pld3290-bib-0048] Toki, S. , Hara, N. , Ono, K. , Onodera, H. , Tagiri, A. , Oka, S. , & Tanaka, H. (2006). Early infection of scutellum tissue with Agrobacterium allows high‐speed transformation of rice. The Plant Journal, 47, 969–976. 16961734 10.1111/j.1365-313X.2006.02836.x

[pld3290-bib-0049] Wang, W. H. , Köhler, B. , Cao, F. Q. , Liu, G. W. , Gong, Y. Y. , Sheng, S. , Song, Q. C. , Cheng, X. Y. , Garnett, T. , Okamoto, M. , Qin, R. , Muelloer‐Roeber, B. , Tester, M. , & Liu, L. H. (2012). Rice DUR3 mediates high‐affinity urea transport and plays an effective role in improvement of urea acquisition and utilization when expressed in *Arabidopsis* . New Phytologist, 193, 432–444. 10.1111/j.1469-8137.2011.03929.x 22010949

[pld3290-bib-0050] Weatherburn, M. W. (1967). Phenol‐hypochlorite reaction for the determination of ammonia. Analytical Chemistry, 39, 971–974.

[pld3290-bib-0051] Webster, G. C. , Verner, R. A. , & Gansa, A. N. (1957). The effect of biuret on protein synthesis in plants. Plant Physiology, 32, 60–61. 10.1104/pp.32.1.60 16654944 PMC540861

[pld3290-bib-0052] Yang, C. , Li, D. , Liu, X. , Ji, C. , Hao, L. , Zhao, X. , Li, X. , Chen, C. , Cheng, Z. , & Zhu, L. (2014). OsMYB103L, an R2R3‐MYB transcription factor, influences leaf rolling and mechanical strength in rice (Oryza sativa L.). BMC Plant Biology, 14, 158 10.1186/1471-2229-14-158 24906444 PMC4062502

[pld3290-bib-0053] Yokotani, N. , Ichikawa, T. , Kondou, Y. , Matsui, M. , Hirochika, H. , Iwabuchi, M. , & Oda, K. (2009). Tolerance to various environmental stresses conferred by the salt‐responsive rice gene *ONAC063* in transgenic Arabidopsis. Planta, 229, 1065–1075. 10.1007/s00425-009-0895-5 19225807

[pld3290-bib-0054] Yoshida, R. , Kanno, A. , Sato, T. , & Kameya, T. (1996). Cool‐temperature‐induced chlorosis in rice plants. I. Relationship between the induction and a disturbance of etioplast development. Plant Physiology, 110, 997–1005. 10.1104/pp.110.3.997 8819872 PMC157800

[pld3290-bib-0055] Yu, N. Y. , Wagner, J. R. , Laird, M. R. , Melli, G. , Rey, S. , Lo, R. , Dao, P. , Sahinalp, S. C. , Ester, M. , Foster, L. J. , & Brinkman, F. S. (2010). PSORTb 3.0: Improved protein subcellular localization prediction with refined localization subcategories and predictive capabilities for all prokaryotes. Bioinformatics, 26, 1608–1615. 10.1093/bioinformatics/btq249 20472543 PMC2887053

[pld3290-bib-0056] Zhao, D. , Sonawane, N. D. , Levin, M. H. , & Yang, B. (2007). Comparative transport efficiencies of urea analogues through urea transporter UT‐B. Biochimica Et Biophysica Acta – Biomembranes, 1768, 1815–1821. 10.1016/j.bbamem.2007.04.010 17506977

[pld3290-bib-0057] Zhao, J. , Wang, S. , Qin, J. , Sun, C. , & Liu, F. (2020). The lipid transfer protein OsLTPL159 is involved in cold tolerance at the early seedling stage in rice. Plant Biotechnology Journal, 18, 756–769. 31469486 10.1111/pbi.13243 PMC7004919

