# Supplementary material for: Overexpression of exogenous biuret hydrolase in rice plants confers tolerance to biuret toxicity
Source: Plant Direct. 2020 Nov 29;4(11):e00290. doi: 10.1002/pld3.290 (PMC7700744; doi:10.1002/pld3.290)
Supplement: Supplementary file 2 — Supinfo S2 [file PLD3-4-e00290-s002.pdf]

SUPPLEMENTAL MATERIALS

Supplemental Methods

Bacterial strain and growth conditions

Rhizobium sp. KaB01 was isolated from agricultural soil using the enrichment culture technique. A 10 g batch of soil that was collected from a farmer's field in Kyoto city was mixed with 50 µmol biuret and put into a 100-mL glass vial. The soil was moistened and incubated at 30ºC for a month. Then, 0.5 g of the incubated soil was suspended into 4 mL of a mineral salt medium containing biuret as a nitrogen source and incubated at 28ºC for 2 days with agitation. A 100 µL aliquot was transferred into a new medium and incubated for 2 more days. Subsequently, this culture was streaked on agar plates with the same mineral salt media, and a colony that grew effectively was isolated. The mineral salt medium contained 10 mmol L^{-1} K_2HPO_4, 3 mmol NaH_2PO_4, 1 mmol L^{-1} $MgSO_4$, trace elements that were described by Pichinoty et al. (1977), 0.25% sucrose as a carbon source, and 0.3 mmol biuret as a nitrogen source. Filter-sterilized biuret solution was added to the medium after autoclaving it.

The taxonomic classification of the isolate was performed by 16SrRNA gene sequencing analysis. Bacterial DNA was extracted from the isolate using Nucleo Spin Tissue (Takara Bio, Shiga, Japan), according to the manufacturer's instructions. A partial fragment of 16SrRNA was amplified using the 10F (5´-GTTTGATCCTGGCTCA-3´) and 800R (5´-TACCAGGGTATCTAATCC-3´) primers and then sequenced under contract (FASMAC Co., Ltd., Kanagawa, Japan). The nearest neighbor strain was identified using the EzTaxon database (Yoon et al., 2017); the partial 16SrRNA sequence of the isolate showed 100% similarity to that of *Rhizobium giardini* bv giardini H152.

Cloning of *biuret hydrolase* from *Rhizobium* sp. KaB01

Biuret hydrolase was cloned based on sequence similarities. First, tBLASTn searches were carried out against *R. giardinii* genes (ARBG01000174) at the National Center for Biotechnology Information (NCBI) database, using sequences of pRL100352 of *R. leguminosarum* bv.viciae 3841(WP_011654379.1; Camelon et al., 2011) as a query. Primers to amplify a region that contained the *biuret hydrolase* homologue were designed based on the sequence of the *R. giardinii* genome (ARBG01000174). A fragment from the DNA of *Rhizobium* sp. KaB01 was PCR-amplified using Blend Taq polymerase (Toyobo, Osaka, Japan) and the designed primers, 5´-GCCCTGTCGATCACGAAATA-3´ and 5´-GGTGTGTCGCGGATCTGG-3´. The amplified fragment was sequenced under contract (Fasmac Co., Ltd., Kanagawa, Japan) and amino acids were characterized.

Expression and purification of a recombinant protein

The open reading frame of the *biuret hydrolase* gene homologue was amplified from the DNA of KaB01 via PCR, using Prime Star polymerase (Takara Bio, Shiga, Japan) and the primers 5′-ATGAAGACACTTTCCAGCGC-3′ and 5′-GAGTCCTGCAGGTGGCAAATGCCTCTCAAGG-3′. The amplified fragment was cloned into the expression vector pMAL-c5X, which contained an amino-terminal maltose-binding protein (MBP) tag. The MBP-fusion protein expressed in *Escherichia coli* was purified using amylose resin, according to the manufacturer's instructions (New England Biolabs Inc., Ipswich, MA, US).

References

Pichinoty F., Mandel M., Greenway B. & Garcia, J.L. (1977). Isolation and properties of a denitrifying bacterium related to Pseudomonas lemoignei. *International Journal of Systematic Bacteriology, 27,* 346-348.

Yoon S.H., Ha S.M., Kwon S., Lim J., Kim Y., Seo H. & Chun, J. (2017). Introducing EzBioCloud: A taxonomically united database of 16S rRNA and whole genome assemblies. *International Journal of Systematic and Evolutionary Microbiology, 67,* 1613-1617.

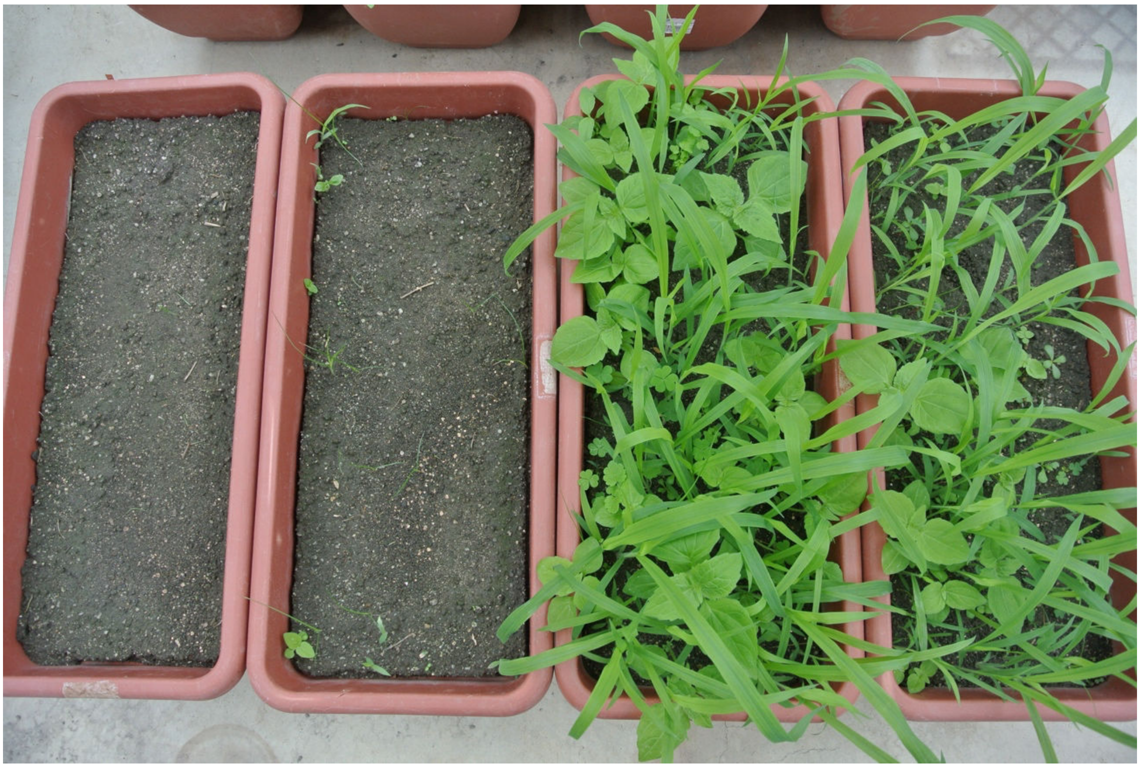

Supplemental Figure S1. Weeds in biuret or urea applied soil. Soil was taken from an experimental field at Kyoto University, air-dried, and sieved. A 6 kg batch of air-dried soil was added into each container (41 x 17 cm^2). Biuret and urea solutions (230 mg N container $^{-1}$) were applied onto the soil surface, and containers were irrigated at appropriate intervals. A photo was taken 24 days after the start of the irrigation process. The two containers on the left contained biuret and the two containers on the right contained urea.

```
KaB01 BH                  MKTLSSAALTGERLSYIEADPYPWPYNGALRPDNTALIIIDMQTDFCGKGGYVDHMGYDL
R. leguminosarum BH       -----MDAMVETNRHFIDADPYPWPYNGALRPDNTALIIIDMQTDFCGKGGYVDHMGYDL
                                 *:.  .  :*:******************************************

KaB01 BH                  SLVQAPIEPIKKVLSAMRAKGYHIIHTREGHRPDLADLPANKRWRSQRIGAGIGDAGPCG
R. leguminosarum BH       SLVQAPIEPIKRVLAAMRAKGYHIIHTREGHRPDLADLPANKRWRSQRIGAGIGDPGPCG
                          ***********:**:****************************************.****

KaB01 BH                  RILTRGEPGWDIIPDLYPLEGEVIIDKPGKGSFCATDLELILNQKRIENIILTGITTDVC
R. leguminosarum BH       RILTRGEPGWDIIPELYPIEGETIIDKPGKGSFCATDLELVLNQKRIENIILTGITTDVC
                          **************:***:***.*****************:*******************

KaB01 BH                  VSTTMREANDRGFECLMLEDCCGATDYGNHLAAIKMVKMQGGVFGAVSNSESLERHLP
R. leguminosarum BH       VSTTMREANDRGYECLLLEDCCGATDYGNHLAAIKMVKMQGGVFGSVSNSAALVEALP
                          ************:***:****************************:****  :* . **
```

Supplemental Figure S2. The alignment of biuret hydrolase proteins from *Rhizobium* sp. KaB01 and *R. leguminosarum* (WP_011654379.1). The alignment was performed using ClustalW 2.1. Asterisks, colons, and periods denote identical residues, conserved substitutions, and semiconserved substitutions, respectively. The conserved active site cysteine residues are boxed.

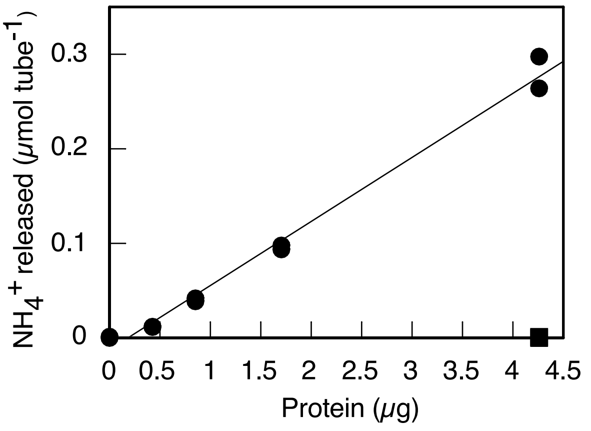

Supplemental Figure S3. Biuret decomposing activity of recombinant KaB01 biuret hydrolase. Maltose binding protein (MBP) fused protein expressed *in E. coli* was purified using amylose resin. Protein solutions were incubated at 30ºC for 15 min with 50 mmol L^{-1} sodium phosphate buffer (pH 8) and 3 mmol L^{-1} biuret. Reactions were stopped by adding 0.5 mol L^{-1} H_2SO_4. The amount of ammonia released from biuret was determined using indophenol blue. Symbols (circles) indicate individual determination. Boxes indicate heat inactivated (100˚C, 10 min) samples.

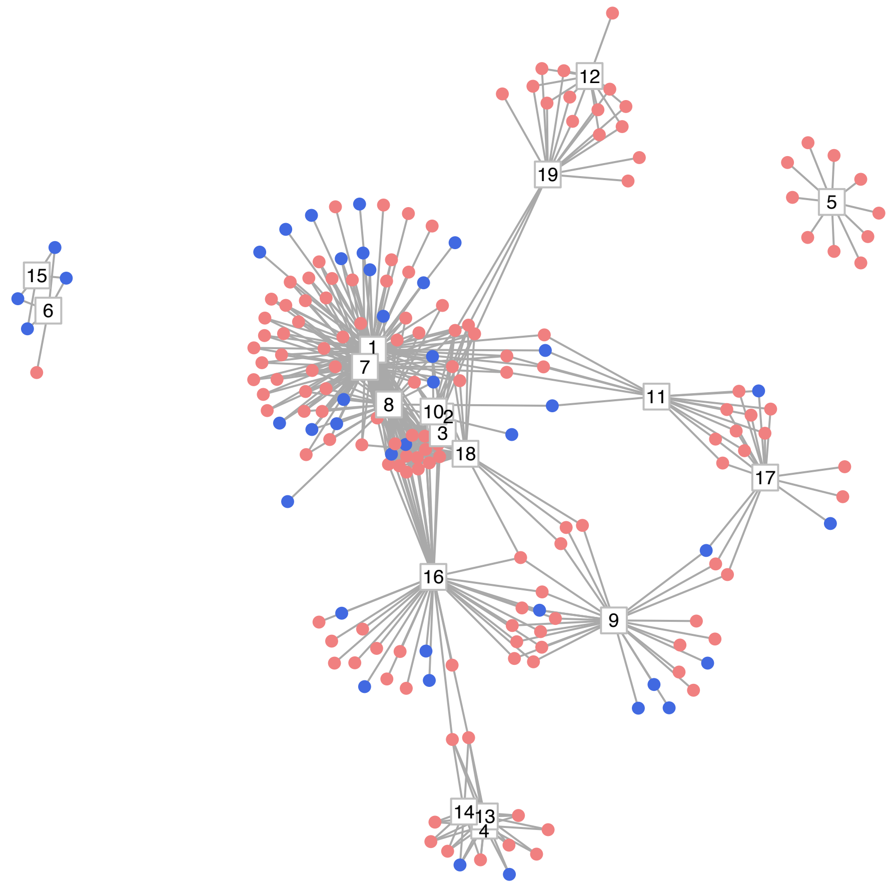

1	oxidation-reduction process	11	electron transfer activity
2	cellular oxidant detoxification	12	glutathione transferase activity
3	hydrogen peroxide catabolic process	13	enzyme inhibitor activity
4	negative regulation of catalytic activity	14	pectinesterase inhibitor activity
5	lipid transport	15	copper ion transmembrane transporter activity
6	copper ion transport	16	extracellular region
7	oxidoreductase activity	17	anchored component of plasma membrane
8	heme binding	18	Phenylpropanoid biosynthesis
9	hydrolase activity, acting on glycosyl bonds	19	Glutathione metabolism
10	peroxidase activity		

Supplemental Figure S4. Interrelationship among GO terms and KEGG pathways overrepresented in DEGs on day 5. Squares indicate GO terms, pink circles indicate up-regulated genes, blue circles indicate down-regulated genes, and lines connecting a circle and a square show that the GO term is associated with the gene.

Supplemental Table 1. Number of GO terms starting with defense response to or response to associated with DEGs. For the Day 5 data, GO terms associated with just a single gene are not shown.

Term id	Term	Day 3	FE	Day 5	FE
GO:0006952	defense response	7	2.0	26	1.9
GO:0042742	defense response to bacterium	4	4.8	5	1.5
GO:0050832	defense response to fungus			3	1.4
GO:0002229	defense response to oomycetes	4	9.3	5	2.9
GO:0006950	response to stress	2	2.4	10	3.0
GO:0009737	response to abscisic acid			10	3.1
GO:0009733	response to auxin	1	1.2	4	1.2
GO:0009617	response to bacterium			3	7.1
GO:0010200	response to chitin			2	9.4
GO:0009409	response to cold			5	2.0
GO:0050826	response to freezing			2	15.7
GO:0009620	response to fungus			2	5.2
GO:0009739	response to gibberellin			2	3.4
GO:0009408	response to heat			6	2.8
GO:0042542	response to hydrogen peroxide			4	4.1
GO:0009416	response to light stimulus			7	2.6
GO:0006979	response to oxidative stress	5	2.8	18	2.6
GO:0000302	response to reactive oxygen species			4	3.3
GO:0009751	response to salicylic acid			2	3.1
GO:0009651	response to salt stress			7	2.1
GO:0009415	response to water			2	6.7
GO:0009414	response to water deprivation	1	1.4	8	2.8
GO:0009611	response to wounding			5	3.6
	others			18	

FE: Fold enrichment. FE = (frequency) / (expected frequency). Values are not significant (Fishers' exact test, adjusted-$p \geq 0.05$).
