# Supplementary material for: Overexpression of exogenous biuret hydrolase in rice plants confers tolerance to biuret toxicity
Source: Plant Direct. 2020 Nov 29;4(11):e00290. doi: 10.1002/pld3.290 (PMC7700744; doi:10.1002/pld3.290)
Supplement: Supplementary file 1 — Supinfo S1 [file PLD3-4-e00290-s001.pdf]

Response to reviewers' comments

Thank you for the careful reading of our manuscript and insightful comments. All of the comments are beneficial for us to improve our manuscript. We have revised our manuscript according to reviewers' comments. One major revision is the addition of microarray-based transcriptome data using rice suspension cells to investigate the responses of rice cells to biuret toxicity. Changes in the text are shown in blue. Please see below for a point-by-point response to reviewers' comments and concerns.

Reviewer #1:

Biuret, a toxic compound to plants, is a minor component of urea fertilizer and an intermediate in s-triazine herbicide biodegradation. The biuret hydrolase is able to catalyze biuret into ammonium. Although the biuret hydrolase has been cloned and studied in several bacteria, however, overexpression of biuret hydrolase in plants has not been studied yet. This manuscript from Ochiai et al. studies the physiological mechanisms underlying biuret toxicity and generates biuret-detoxifying rice by overexpression a biuret hydrolase from Rhizobium sp. KaB01. The 15N-labelled biuret was used to evaluate biuret uptake in rice plants. The results indicated that biuret could reach sub millimolar concentrations in rice plants, and overexpressing a bacterial biuret hydrolase increased tolerance to biuret toxicity. Authors proposed that biuret could act as a slow-release N fertilizer and weed controller. Overall, the manuscript is well written, questions posed by the authors are new, and all figures are well presented. Nevertheless, there are some points that should be addressed and these are outlined below:

1. One of objectives of this study was the investigation of physiological mechanism of biuret-induced toxicity on rice. However, only plant height and chlorophyll content were examined. Authors should provide more data to reveal the toxic effect of biuret on rice, such as root growth, the accumulation of reactive oxygen species (H2O2 and superoxide), photosynthetic efficiency like Fv/Fm.

Thank you for the valuable comments. Root growth decreased with increasing biuret concentration (please see Fig. 1b), though we do not show quantitative data. As you pointed out, the data to reveal the toxic effect of biuret on rice was insufficient in our original manuscript. We added some experiments using rice suspension cells (Section 3.6). Please see our revised manuscript.

2. Treatment of biuret inhibits rice growth and induces albino leaves. Author should discuss the possible reason.

We appreciate the suggestion. We presume that retarded growth in mild biuret toxicity that did not kill rice plants might be due to a trade-off with adaptive responses. The albino leaves were similar to those often found in rice seedlings exposed to cold or sudden high light intensity. Biuret injury may cause similar downstream reactions that are brought by such stresses. We added discussions on these points (page 21-23).

3. Figure legend of Table 1 was easy to be confused. It seems only two plants were grown in a 40-mL vial and used for analysis. If it is true, I would suggest more plants (at least 3 plants).

The uptake experiment was performed in triplicate. Two seedlings were incubated in each of three vials. Then, the two seedlings in a vial were analyzed as one sample. We revised the legend. Thank you.

4. Two independent overexpressing lines with the same number (B2-3-3-3) were showed in Table 2. Author should revise it. In addition, mean values of plant height in WT and transformants are very close after biuret treatment. Although t-test showed a significant difference after biuret treatment in two transformants, the mean values with biuret treatment were located inside the range of mean{plus minus}SD of WT. Therefore, a multiple range test is suggested as the post hoc test to clearly demonstrate the significance among all tests.

We applied two-way ANOVA with Tukey-Kramer posthoc test on the data in Table 2, as suggested. Descriptions in the text were revised to the reflect results of the tests. Additionally, we converted the original Table 2 into the new Figure 6.

5. Figure 2 showed the RNA expression levels of biuret hydrolase in transgenic rice by real-time PCR analysis. The values showed in the figure were means of two technical replicates. This data is suggested to have three repeats and show as mean{plus minus}SD.

We added error bars in Fig. 2. On the whole, the values of the duplicate were in good agreement. We could not repeat the determination because the T_0 plants have already died.

6. B3-9-1 line has release a great amount of ammonium as compared to B2-3-3-3. The real-time PCR data showed these two lines have similar RNA accumulation. Authors should discuss the inconsistence. In addition, the ammonium level of B2-3-3-3 is similar with WT, therefore, determination of biuret hydrolase activity is strongly suggested.

We appreciate your suggestion. We have already determined biuret hydrolase activity (please see Figure 3). As the expression level of mRNA increases, so does the expression level of protein, but generally, the correlation between them is rough. We suppose it is the reason for the inconsistency. Another possible reason is the underestimation of the enzyme activity in B-2-3-3-3 as biuret hydrolase is easily deactivated. However, the B3-9-1 seedlings appear to have greater tolerance than other over-expressing lines in our currently ongoing culture experiments under sever biuret toxicity. Thus, we suppose that the biuret hydrolase activity of this line is higher than other lines.
We added some descriptions to the text (Page 15, Line 306-308).

7. In Figure 4, overexpression of a bacterial biuret hydrolase increased tolerance to biuret toxicity in rice seedlings. It will be nice to know the yield elements of overexpressed rice plants to demonstrate the biuret could act as a slow-release N fertilizer without yield penalty.

Thank you for your comment. We are currently performing a long-term growth experiment. We would like to publish the result in our next paper.

8. Typo: P7, line 131: 35s should be changed to "35S"

We corrected it. Thank you.

Reviewer #2:

The manuscript from Ochiai et al 'Overexpression of exogenous biuret hydrolase in rice plants confers tolerance to biuret toxicity' is a solid study of the transgenic expression of bacterial biuret hydrolase in rice, which they claim confers resistance to biuret and that these plants can potentially now use biuret as a fertiliser. Morever, they claim that 15-N studies provide insight into the mechanism of toxicity of biuret.

The biuret hydrolase used in this study was isolated from a freshly isolated bacterial strain - it's unclear why the authors chose to do this as there are previously characterised biuret hydrolases in the literature and gene synthesis is almost certainly less expensive than isolated a new sequence (> 90% identical to known sequences).

Thank you for your suggestion. We would like to try synthetic genes in our future occasions. In regards to the current study, we have already cloned the gene and generated transgenic rice lines. When we started our experiment in 2012, we thought several unknown enzymes could catalyze biuret degradation. That is the reason why we started with bacterium isolation. Fortunately, we could smoothly isolate *Rhizobium* spp.. It may be because we took the soil from a farmer’s field cultivated under high fertilizer input. Subsequent experiments took time.

The recent literature regarding this family of enzyme ought also be cited, including the recent study from Aukema et al in Appl Env. Microbiol this year, and the review by Esquirol et al in Adv. Microbial Physiol. (also this year). The rationale for the authors' approach ought to be provided.

We appreciate the suggestion. We cited the literature (Page 4, second paragraph).

Regardless, of its source the functional expression of biuret hydrolase in transgenic plants is clearly demonstrated, as is a level of resistance to biuret conferred by the presence of the enzyme. The claim that biuret may act as a fertiliser when biuret hydrolase is expressed is less well supported by the data - there appears to be a very small growth advantage (which is supported by statistical analysis). The concept of using triazine-catabolism related enzymes to allow use of triazines and related compounds as a Nitrogen fertiliser is suggested in the recent review by Esquirol et al (Adv. Microbial Physiol 2020) - which should be cited.

Thank you for your suggestion. We cited the literature (Page 4, Line 79).

In our experiments, the hydroponic culture solution contained enough amount of nitrogen as $(NH_4)_2SO_4$. Nitrogen was not a major growth-limiting factor in the experimental condition. Therefore, the small growth advantage seems to be natural. Besides, the results of the ^{15}N-biuret uptake experiment showed that biuret uptake was increased in the transgenic lines.

While the wild-type rice plants' growth was reduced due to biuret injury, the transformed strains' growth seemed slightly increased. Thus, we suppose this plant material is suitable for

examining the fertilizer effect of biuret. We are currently conducting a long-term cultivation experiment to test the fertilizer effect of biuret.

Unfortunately the 15-N biuret uptake studies were not informative about the mechanism of biuret toxicity, they merely confirmed that biuret is accumulated in plant tissues. I would suggest that comments regarding insights into the physiological mechanism be removed from the introduction and elsewhere, as this study does not provide such insights.

We appreciate the comments. We believe the determination of uptake is essential for understanding the toxic and nutritional effects of biuret. However, we agree with you that data to reveal the toxic effect of biuret was insufficient in our original manuscript. We removed the following two sentences from the last paragraph of the introduction: "The principal aim of our study is to elucidate the physiological mechanisms underlying biuret toxicity. Additionally, we intend to confer biuret-detoxifying ability to crop plants." Further, we add data on transcriptome analysis in rice suspension cells (Section 3.6). Please see our revised manuscript.

We wish to thank you again for the valuable comments.
